# When Not to Operate on Acute Cases—A Surgeon’s Perspective on Rapid Assessment of Emergency Abdominopelvic Computed Tomography

**DOI:** 10.3390/jimaging9100200

**Published:** 2023-09-28

**Authors:** Catalin Alius, Dragos Serban, Laura Carina Tribus, Daniel Ovidiu Costea, Bogdan Mihai Cristea, Crenguta Serboiu, Ion Motofei, Ana Maria Dascalu, Bruno Velescu, Corneliu Tudor, Bogdan Socea, Anca Bobirca, Geta Vancea, Denisa Tanasescu, Dan Georgian Bratu

**Affiliations:** 1 Faculty of Medicine, Carol Davila University of Medicine and Pharmacy Bucharest, 020021 Bucharest, Romania; catalin.alius@umfcd.ro (C.A.); bogdan.cristea@umfcd.ro (B.M.C.); crenguta.serboiu@umfcd.ro (C.S.); ion.motofei@umfcd.ro (I.M.); ana.dascalu@umfcd.ro (A.M.D.); corneliu.tudor@umfcd.ro (C.T.); bogdan.socea@umfcd.ro (B.S.); anca.bobirca@umfcd.ro (A.B.); geta.vancea@umfcd.ro (G.V.); 2 Fourth General Surgery Department, Emergency University Hospital Bucharest, 050098 Bucharest, Romania; 3 Faculty of Dental Medicine, Carol Davila University of Medicine and Pharmacy Bucharest, 020021 Bucharest, Romania; 4 Department of Internal Medicine, Ilfov Emergency Clinic Hospital Bucharest, 022104 Bucharest, Romania; 5 Faculty of Medicine, Ovidius University Constanta, 900470 Constanta, Romania; daniel.costea@365.univ-ovidius.ro; 6 General Surgery Department, Emergency County Hospital Constanta, 900591 Constanta, Romania; 7 Department of General Surgery, Emergency Clinic Hospital “Sf. Pantelimon” Bucharest, 021659 Bucharest, Romania; 8 Faculty of Pharmacy, Carol Davila University of Medicine and Pharmacy Bucharest, 020021 Bucharest, Romania; 9 Clinical Hospital of Infectious and Tropical Diseases “Dr. Victor Babes”, 030303 Bucharest, Romania; 10 Department of Nursing and Dentistry, Faculty of General Medicine, ‘Lucian Blaga’ University of Sibiu, 550169 Sibiu, Romania; denisa.tanasescu@ulbsibiu.ro; 11 Faculty of Medicine, University “Lucian Blaga”, 550169 Sibiu, Romania; dan.bratu@ulbsibiu.ro; 12 Department of Surgery, Emergency County Hospital Sibiu, 550245 Sibiu, Romania

**Keywords:** abdominal computed tomography, algorithm, abdominal pain, acute appendicitis, acute cholecystitis, bowel occlusion, acute pancreatitis

## Abstract

Clinical problem solving evolves in parallel with advances in technology and discoveries in the medical field. However, it always reverts to basic cognitive processes involved in critical thinking, such as hypothetical–deductive reasoning, pattern recognition, and compilation models. When dealing with cases of acute abdominal pain, clinicians should employ all available tools that allow them to rapidly refine their analysis for a definitive diagnosis. Therefore, we propose a standardized method for the quick assessment of abdominopelvic computed tomography as a supplement to the traditional clinical reasoning process. This narrative review explores the cognitive basis of errors in reading imaging. It explains the practical use of attenuation values, contrast phases, and windowing for non-radiologists and details a multistep protocol for finding radiological cues during CT reading and interpretation. This systematic approach describes the salient features and technical tools needed to ascertain the causality between clinical patterns and abdominopelvic changes visible on CT scans from a surgeon’s perspective. It comprises 16 sections that should be read successively and that cover the entire abdominopelvic region. Each section details specific radiological signs and provides clear explanations for targeted searches, as well as anatomical and technical hints. Reliance on imaging in clinical problem solving does not make a decision dichotomous nor does it guarantee success in diagnostic endeavors. However, it contributes exact information for supporting the clinical assessments even in the most subtle and intricate conditions.

## 1. Introduction

“Clinical reasoning is as old as humanity”, and the process of formulating a diagnosis has always been correlated with the scientific, social, and technological “status quo” [1]. When imaging modalities were scarce and less sophisticated, impressions were based merely on clinical signs and wily inferences. Today, at the core of clinical reasoning lies the notion of differential diagnosis, which was first introduced in an official capacity by William Osler et al. [2]. This concept has been embraced by modern medicine and incorporated into all methods that deal with the systematic assessment of diseases. In acute cases, when a rapid decision must be made and the masquerading nature of some conditions causes clinical conundrums, a systematic assessment of abdominopelvic computed tomography (CT) could prove to be a valuable supplement during the clinical reasoning process [3]. This idea is supported by an increase in emergency CTs, which are most likely to aid the best and most prompt case-management decisions [4]. This study offers a surgeon’s perspective on diagnosing acute cases based on clinical and radiological findings by merging these two entities into an assessment algorithm. In general, solving a clinical problem reverts one to basic cognitive processes involved in critical thinking, such as hypothetical–deductive reasoning, pattern recognition, and compilation models [5,6,7]. This article aims to provide a supplementary tool to clinical reasoning for clinicians involved in the management of acute cases in order to increase their diagnostic skills, prognostic predictions, and overall management of their patients.

## 2. The Algorithm for Clinical Decisions Concerning Abdominal Pain

**Figure 1:**
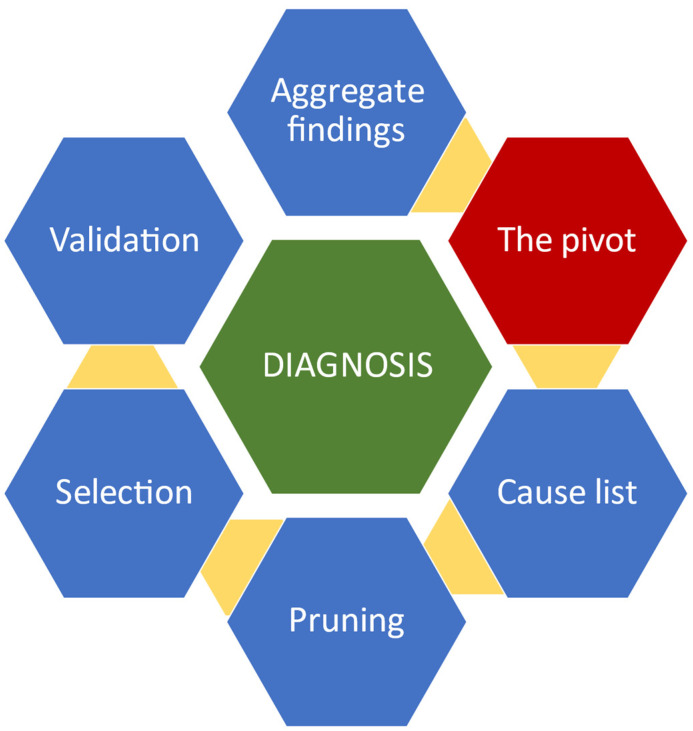
Schema of the clinical problem-solving model based on Eddy’s and Clanton’s concept.

A few decades ago, Eddy and Clanton [8] proposed a six-step clinical problem-solving model (CPS) for reaching final diagnoses (Figure 1).

The clinician gathers elementary cues, such as clinical signs, the history of the disease, test results, etc., into blocks of information that allegedly share a common physiopathological background. This aggregate of cues forms the Pivot, which has the role of reducing the complexity of the clinical problem. A list of differential diagnoses is then developed. These are compatible with the Pivot but are matched with all of the findings of the case, one by one, during Pruning. When more diagnoses match the Pivot and the available case data, additional tests or cues are sought until a definitive unique diagnosis is selected. Finally, this is validated against all of the findings of the case. Pattern recognition is vital during the development of the Pivot, and deductions are necessary to develop hypotheses during the ensuing steps of the CPS process.

However, when clinicians recognize a typical pattern, they might fall for the mirage of the first lesion, unwisely skipping the Pruning, Selection, and Validation, and increasing the risk of advancing an incorrect diagnosis.

If a Pivot cannot be established, the natural course of action will be the formulation of a hypothesis by employing basic pathological mechanisms and deductions. In this review, we focus on searching for patterns when reading abdominopelvic CTs, radiological cues, and technical modifications of radiological examinations to enhance their accuracy and their relevance in achieving rapid and correct diagnosis in cases of acute undifferentiated abdominal pain.

## 3. The Need for Standardization of Search Patterns in Emergency Abdominopelvic CT

Multiple-target visual search is the mainstay of abdominopelvic CT interpretation. Unfortunately, it is affected by a phenomenon that was described by Smith et al. [9] almost six decades ago and was recently renamed by Adamo et al. [10] as the Subsequent Search Miss (SSM). Confirmation bias, perceptual errors, and depletion of cognitive resources are the main culprits causing these errors.

In a recent paper by Kliever et al. [11], the authors brought forward the issue of the “idiosyncratic” character of searching CT images, which is caused by the diversity of the searching methods employed by clinicians who report imaging results. A systematic examination could reduce the search errors but will not influence the recognition errors, which are based on knowledge (not attention). Another contributor to SSMs is the prevalence of lesions or findings. As demonstrated by Wolfe et al. [12], rare items are frequently missed because of a phenomenon called “vigilance decrement” [13]. As a result of low rates of exposure to certain pathologies, the lower their prevalence, the higher the risk of a false negative result. This can be improved by practicing with curated image stacks that keep the memory trained and maintain a minimal degree of awareness [14].

**Figure 2:**
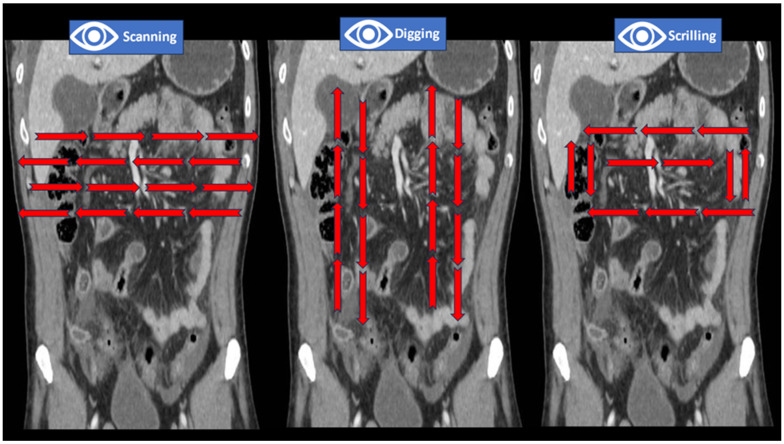
Techniques for multiple-target searching during CT examination. The red arrows show the direction of the visual search pattern.

The influence of clinical information on reporting was demonstrated in a prospective blinded study that analyzed the results written before and after clinical input. Inconsistent clinical details have a detrimental effect, while accurate information improves the quality of a report [15,16,17,18]. When expert readers assess CTs, they use three techniques: drilling, scanning, and “scrilling”. Drilling refers to evaluating a stack of images by using small visual corridors that are parallel to each other and allow in-depth visualization of structures. Scanning implies using slices rather than corridors and is more suitable for organs that span horizontally, such as the pancreas. “Scrilling” is a mixed technique combining in-depth analysis with scanning and helps assess sections containing organs of different shapes and densities, such as the abdomen and pelvis [16] (Figure 2).

**Figure 3:**
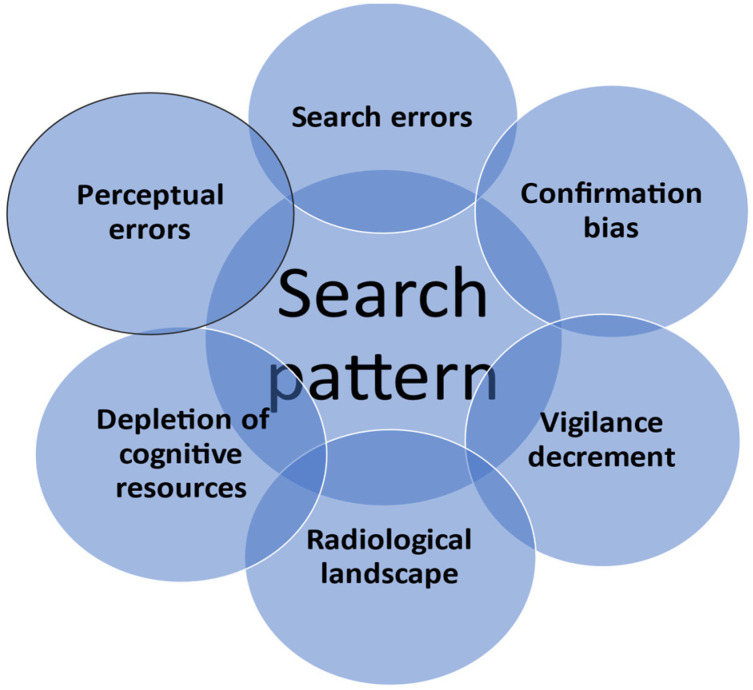
Cognitive processes involved in errors of reading imaging.

A prospective study by Parag et al. [19] demonstrated that the interpretation of emergency CTs by surgeons for noncritical findings in emergency trauma patients was comparable to that of radiologists; therefore, all surgeons involved in acute cases should be equipped with appropriate training in reading images, especially in institutions with off-site CT services. Norman et al. [20] stated that clinical reasoning is better and faster with input from radiological studies, hence the need for a surgeon’s perspective when interpreting CTs for decisions pertaining to patients with undifferentiated acute abdominal pain (Figure 3).

## 4. Map Charting, Image Modulators, and Discovery of Cues

**Figure 4:**
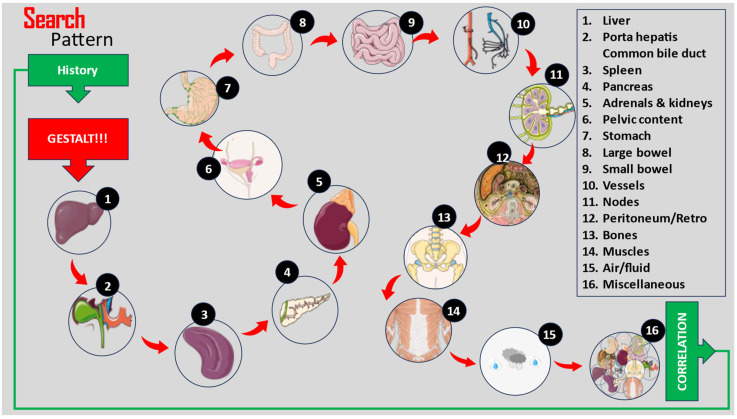
Steps for the rapid assessment of an abdominopelvic CT.

Acute abdominal pain accounts for up to 10% of all presentations at emergency departments [21,22]. Many patients will receive a CT due to the implementation of this imaging exam as an early diagnostic tool, but less than half of these scans are more or less superfluous [23,24]. This is why the endeavors of a surgeon who tries to read a CT scan must be mirrored by a radiologist, who, in turn, should seek the appropriate history before starting to interpret the images. We devised a practical approach for the quick assessment of any abdominopelvic CT images based on 16 steps through which clinicians search for radiological cues to support their clinical suspicions (Figure 4).

The role of a holistic first look is advocated by numerous authors who claim that by glancing over the scan, the brain can recognize patterns by employing neural network pathways based on the concepts of proximity, similarity, continuity, closure, and grouping [25,26,27,28]. Some could argue that the same principles of perception are involved in errors of searching, but the global look should not replace a systematic segmental approach. CT images are visual transformations of tissue radiodensity translated into tones of gray.

The human eye is incapable of discerning discrete increments in tonal intensity, unlike computers [29]. Therefore, quantitative measurement of such tonalities can be achieved by employing a measurement scale that is read and interpreted by a machine [30]. By convention, water is attributed 0 Hounsfield units, air is attributed −1000 units, and bone and metal are attributed +1000 units. In a simplified version, one should understand that the denser the tissue is, the higher the number of Hus displayed and vice versa. Examination windows, which act similarly to low- and high-pass filters, are derivatives of the quantitative measurement of radiodensity. The interval between some arbitrarily chosen limits is called the window’s width, and the midpoint of the range of the measurements displayed is called the level or the center of the window. Increasing the window will decrease the image’s contrast, while narrowing it will do the opposite. Windowing with a large width is useful when the reader wants to see air, while a narrow width helps characterize parenchymatous lesions.

**Figure 5:**
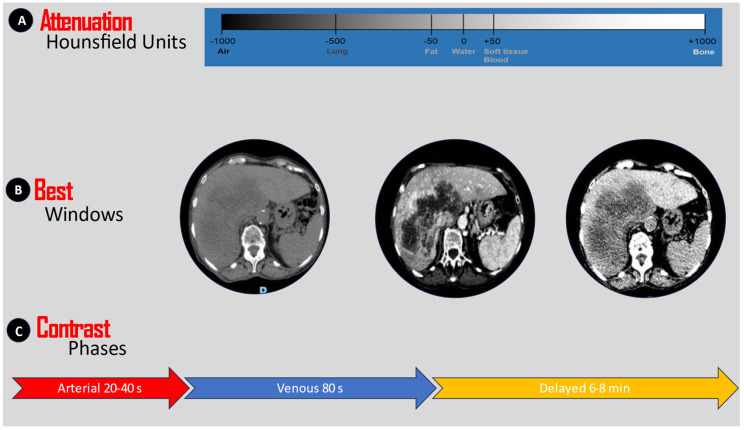
The ABC of lesion enhancement during CT interpretation ((A) attenuation, (B) best window, (C) contrast phases).

Contrast-enhanced CT implies the acquisition of images after the administration of oral or/and IV contrast. Although the administration of oral contrast is debatable, and no consensus on its use has been reached, IV contrast is recommended for most abdominopelvic acute conditions [31,32,33]. Without mentioning the subtleties and subphases of contrast administration, there are, in general, three moments for image acquisition that should be reviewed by the clinician: the arterial phase, the venous phase, and the delayed phase. All these are dynamic representations of tissular perfusion and urine excretion. During the first 40 s, the contrast enhances the arteries and well-vascularized structures; during the venous phase, paucivascular areas become visible, and during the delayed phase, kidney excretion and urinary structures are enhanced. Although surgeons do not devise examination protocols, they must recognize the three main phases—the arterial, venous, and delayed phases—of a contrast-enhanced scan, their utility in delineating anatomical structures, and the use of examination windows for grayscale adjustments (Figure 5).

**Figure 6:**
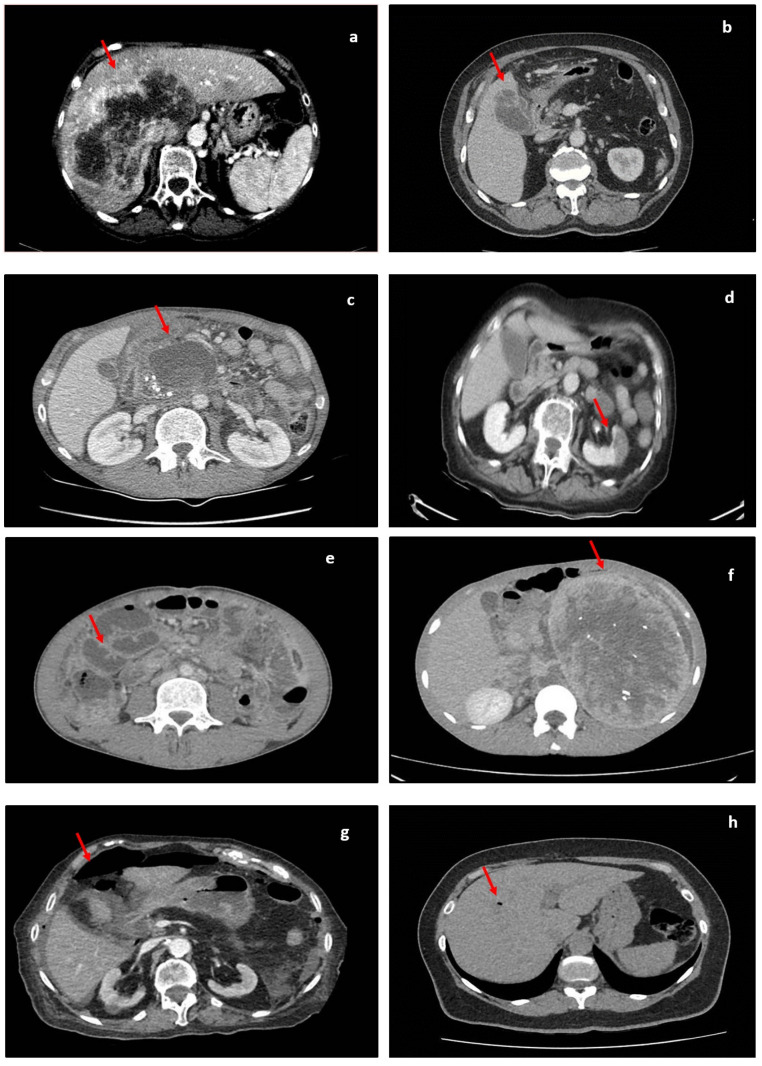

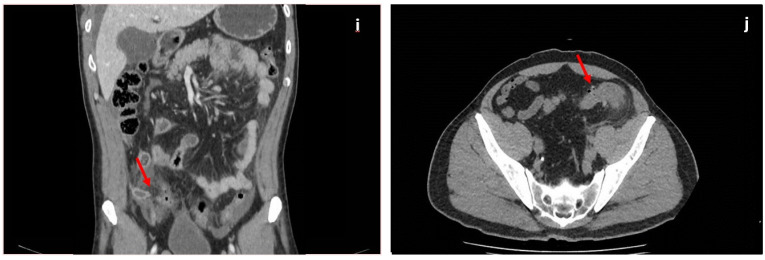
Different CT findings in abdominopelvic pathologies. Lesions are indicated by red arrows. (**a**) Image showing a large liver lesion; (**b**) image showing fat stranding and gallbladder wall discontinuity; (**c**) image showing a large pancreatic pseudocyst; (**d**) image showing left-kidney hypoattenuation during the venous phase, which is suggestive of ischemic changes; (**e**) image showing multiple distended small-bowel loops; (**f**) image showing a large left-retroperitoneal mass; (**g**) image showing a pneumoperitoneum; (**h**) aerobilia; (**i**) acute appendicitis coronal (fat stranding around the caecum and an inflamed appendix); (**j**) image showing perisigmoidian fat stranding and air pockets in acute diverticulitis.

We grouped these instruments for image modulation under the practical name of “the ABC of lesion enhancements”, and we believe that, together with anatomical concepts and the use of clinical patterns, these form a triad for the correct interpretation of CT images by non-radiologists. Clinicians use a mental catalog of normal and abnormal findings when searching for lesions; hence, the following 16 steps should be approached in order during interpretation (Table 1, Figure 6).

## 5. Merging Clinical Pathways with Radiological Cues

**Figure 7:**
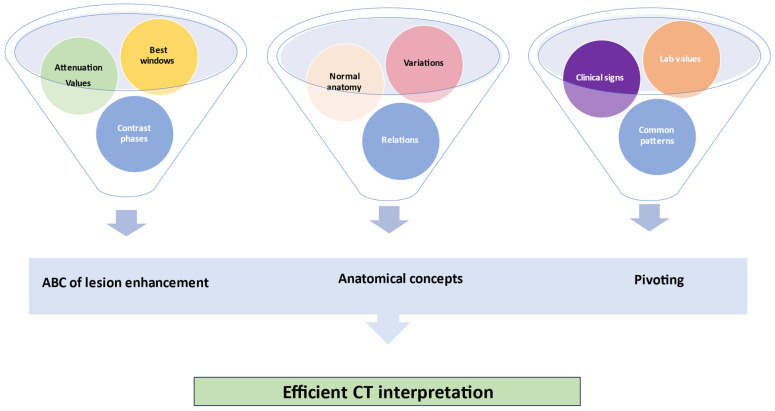
The triad of efficient CT interpretation: the ABC of lesion enhancement (A—attenuation, B—best window, C—contrast phases), anatomical concepts, and pivoting.

Over recent decades, CPS in the acute abdomen has shifted from a purely clinical undertaking to an intricate and multidisciplinary process [34]. The universal onus of reducing morbidity and mortality through judicious operative and nonoperative decisions is time-dependent and cannot be implemented without the use of advanced imaging supplements, such as CT [35]. Because many institutions do not maintain on-call services, and because studies have shown the acceptable accuracy of scan interpretation by properly trained non-radiologists, we believe that a decisional algorithm combining clinical pathways with radiological findings might be beneficial for the rapid assessment of cases presenting with acute abdominopelvic pain [36]. Pattern recognition and identification of clinical vignettes coupled with the use of the principles of the ABC of lesion enhancement and the application of anatomical concepts form the scaffolding triad for a combined clinical–radiological algorithm for rapid assessment and optimal CPS (Figure 7).

### 5.1. Acute Appendicitis

Acute appendicitis (AA) carries a lifetime risk of up to 9% in Western countries but remains difficult to diagnose based only on clinical criteria [37]. The Alvarado score showed unsatisfactory sensitivity and specificity mainly because of the large number of differential diagnoses associated with AA [38,39]. If left untreated, it leads to serious complications, such as perforation, hematogenous spread, abscess formation, and progression of septic syndrome, hence the urgency for rapid diagnosis and treatment. Pelin et al. [40] demonstrated that USS is not a satisfactory modality in obese patients, in those with atypical anatomical positioning of the appendix, or in those with established local complications. CT offers superior specificity and sensitivity, and when coupled with judicious clinical reasoning, it can reveal a diagnosis in almost all cases [41]. Traditional clinical and laboratory signs include right iliac fossa (RIF) pain, nausea/vomiting, anorexia, fever, and elevated white blood cell (WBC) count. Radiological signs include dilatation of the appendiceal lumen, a thickened wall with contrast enhancement, and peri-appendiceal fat stranding, fluid, or a localized ileus in the RIF. A pivot consisting of pain, inflammatory changes, and anorexia might be found in other conditions mimicking AA, such as mesenteric adenitis, Crohn’s ileitis, infections with Yersinia or Campylobacter, torsion of epiploic appendages, or tubo-ovarian abscesses [42]. Very few of these conditions require urgent intervention, with some even being exacerbated by surgery [43]. This underscores the value of radiological signs when establishing a definitive diagnosis. Radiological changes will always correlate with anatomical concepts and clinical findings. This is why all clinicians should validate their diagnoses with radiological cues. How can someone differentiate between Meckel’s diverticulum and an ovarian abscess based only on clinical grounds? Why would someone operate on a 19-year-old with mesenteric adenitis or a young woman with a twisted appendix epiploica?

### 5.2. Acute Pancreatitis

Acute pancreatitis is a potentially fatal condition with an increased incidence over the last 50 years [44,45]. Its varied etiology includes alcohol consumption, metabolic disturbances, exposure to certain drugs and toxins, and autoimmune conditions, with the most common cause being represented by gallstones [46]. Unlike four decades ago, the role of surgery in acute pancreatitis is now reserved for cases with compartment syndrome, infected collections of pseudocysts, fistulae, or vascular complications [47]. Diagnostic laparotomies for pancreatitis are now obsolete because imaging can provide sufficient data to confirm a diagnosis even in a situation in which enzymatic levels are less than three times their normal values [48]. The detection of complications such as abdominal distension and progressive tenderness, fever, elevation of the white blood cell count, or any acute changes in clinical condition by means of CT has further reduced the number of unnecessary surgeries [49]. In general, for clinicians, reading imaging is sufficient to be able to differentiate between a perforated ulcer and acute pancreatitis in its early phase, but during the clinical progression of the condition, more subtle changes have to be recognized. During the first week of the clinical progression of the pancreatitis edema of the gland, enlargement and fading of the usual morphology might not be associated with fat stranding or necrosis [50]. Imaging confirms the diagnosis and allows one to ascertain the severity and the etiology of pancreatitis, allowing for prompt and specific treatment. In later stages, it provides identification of complications and facilitates timely intervention.

### 5.3. Bowel Obstruction

Bowel obstruction is common and represents about 20% of admissions with acute abdominal pain in surgical departments [46]. This is one of the few diagnoses for which effective management and avoidance of unnecessary surgery depend on accurate diagnosis and confirmation of etiology [47]. When caused by adhesions, neoplasms, herniations, inflammatory bowel diseases, intussusception, volvulus, or foreign bodies, it is categorized as mechanical, and when there is no demonstrable transition point or mechanical cause, it is categorized as functional (ileus) [47,48,51]. Although the diagnosis of bowel obstruction may be established by using clinical findings, such as absolute constipation, abdominal distention, and vomiting, finding the etiology is crucial for avoiding surgery. A plain abdominal radiograph demonstrating dilated bowel loops with fluid levels is not contributory to the cause; therefore, a complementary modality is indicated [46].CT is the technique of choice, as it has an accuracy of more than 95% [52,53,54,55]. In cases of bowel obstruction, CT reveals dilated/collapsed bowel loops, transition points (beak signs), intestinal ischemia, or a swirl sign. Many patients are treated with non-surgical management, but evidence of free air, free fluid, pneumatosis, or sepsis requires urgent surgical treatment [56,57,58].

### 5.4. Acute Cholecystitis

Biliary stones are highly prevalent, especially in Western populations, but almost 70% of the cases remain asymptomatic [44]. Although acute cholecystitis is considered the most frequent pathology admitted to surgical departments, and delays in treatment can lead to the life-threatening complications, its management and diagnostic criteria are still debatable in spite of the multiple guidelines that have been published [59,60]. According to the 2016 WSES guidelines on acute calculous cholecystitis, abdominal ultrasonography is considered the first choice of imaging investigation, but some studies have demonstrated that ultrasonography alone has a high rate of false negative results for acute cholecystitis [60,61,62,63].

Right upper abdominal pain, fever, vomiting, signs of peritoneal irritation, moderate leukocytosis, and ultrasonography findings are helpful but not specific, and they barely help to differentiate other pathologies that do not have indications for surgical treatment, such as hepatitis, peptic ulcer disease, pancreatitis, primary hepatic abscess, gastroenteritis, and urinary tract infection [59,60,61,62].

In case of doubts regarding the diagnosis or the presence of complications, a CT scan can be performed, as this is considered the most sensitive investigation available in ED [62,64]. CT findings are not specific; therefore, diagnostic criteria such as gallstones, a thickened gallbladder wall, pericholecystic fluid collections, and subserosal edema though pericholecystic inflammatory changes are often usual findings [64]. Using clinical signs and the diagnostic criteria in which acute cholecystitis is defined as the presence of three or more classic CT findings, clinicians have increased confidence in their diagnoses, which is crucial in deciding on an operative versus a conservative approach.

## 6. Conclusions

Reliance on imaging in CPS does not make a decision dichotomous nor does it guarantee success in diagnostic endeavors. However, it contributes exact information to support clinical investigations for even the most subtle and intricate conditions. A borderline Alvarado score might be in favor of surgery due to a tiny amount of fat stranding around the caecum. Similarly, but on a bolder note, multiple distended bowel loops showing air–fluid levels could point towards a narcotic bowel rather than a mechanical obstruction in a young addict with uniformly ubiquitous distension and no transition point. We believe that in times of increased need for urgent clinical decisions, all practicians should be equipped with CT interpretation skills. A standardized approach to charting a rigorous and systematic search pattern, accompanied by sound clinical reasoning, is the modern prerequisite for a safer practice.

## Figures and Tables

**Table 1:** Algorithm for the systematic reading and interpretation of an abdominopelvic CT.

Step	Salient Features and Pearls	Anatomical Concepts	Attenuation Values	Best Windows	Contrast Phases
Step 1: Liver	Size, shape, morphology of the parenchyma, duct dilatation, veins, and arteries	Liver segmentation, variations in the portal vein branching	Cystic lesions and hypo- and hyper-attenuating lesions	Use appropriate soft-tissue windows to enhance parenchymatous lesions and aeroportia by using the lung window	Examine during all contrast phases
Step 2: Porta Hepatis, Gallbladder, and CBD	Size (long and short axes), contour, and features of the gallbladder wall (edematous, discontinued, heterogeneity), presence of stones, adenomyomatosis, fluid around gallbladder	Anatomical variations, presence, and character of the dilatation of the biliary tree (choledochal cysts, distal narrowing, wall homogeneity)	Assess for the presence of air in the biliary tree and the periportal and periductal oedema	Use appropriate soft-tissue windows to enhance parenchymatous lesions. Change to lung for the detection of air	Examine during all contrast phases
Step 3: Spleen	Size (best in coronal), contour, and integrity of the capsule, fluid around the spleen, and proximity of lesions	Accessory spleens, splenic hilum	Cystic lesions, hypo- and hyperattenuating lesions, fluid attenuation values, and calcifications	Use appropriate soft-tissue windows to enhance parenchymatous lesions	The spleen is inhomogeneous during the arterial phase and homogenizes during the venous phase. Search for filling defects and correlate appearance with phase
Step 4: Pancreas	Pancreatic contour (diffuse enlargement and heterogeneous attenuation), presence of edema, peripancreatic fluid, hypo- and hyper-attenuating masses, duct dilatation/cut-off, acute peripancreatic collections, acute necrotic collections, pseudocysts, walled-off necroses, atrophy	Anatomical variations (cystic lesions, divisum, or annular pancreas)	Intra- and/or peripancreatic presence of air, reticular stranding of the surrounding fat, calcifications	Use appropriate soft-tissue windows to enhance parenchymatous lesions. Change to lung for the detection of air	Examine during all contrast phases. For suspicion of acute pancreatitis, a pancreatic arterial phase scan is required (acquired 45 s after the injection starts)
Step 5: Adrenals and kidneys	Size (long and short axes), shape, presence of macroscopic fat, nodules, thickening, hypo- and hyper-attenuation, atrophy, hydroureter and hydronephrosis, cysts, perinephric fluid, abscess	Linear or V-/Y-shaped; ectopic, fused/horseshoe kidney, corticomedullary differentiation	Calcifications, stones, perinephric stranding, gas	Use appropriate soft-tissue windows to enhance parenchymatous lesions	Examine during all contrast phases. NB: washout of contrast (rapid/slow)
Step 6: Pelvic content	Size (long and short axes), contour, presence of masses, cysts, free fluid, abscess	Anatomical variations, presence of genital organs, cysts	Fat stranding, calcifications	Use appropriate soft-tissue windows to enhance parenchymatous/intramural lesions. Change to lung for the detection of air	Examine during all contrast phases
Step 7: Stomach	Size (long and short axes), shape (dilation, stenosis), wall thickening, masses, previous surgeries	Anatomical variations, diverticulum, cysts	Assess for presence of free air/fluid, foreign bodies	Use appropriate soft-tissue windows to enhance parenchymatous/intramural lesions. Change to lung for the detection of air	Examine during all contrast phases
Step 8: Large bowel	Bowel loop diameter (dilated/decompressed), wall thickening, pneumatosis, stricture, diverticula, bowel content (fluid, solid, stool), mass lesions, free fluid	Anatomical variations	Free air, fat stranding, foreign bodies, abnormal density (hemorrhage), +/− oral contrast	Use appropriate soft-tissue windows to enhance parenchymatous/intramural lesions. Change to lung for the detection of air	Examine during all contrast phases
Step 9: Small bowel	Bowel loop diameter (dilated/decompressed), wall thickening, pneumatosis, strictures, diverticula, mass lesions, free fluid	Anatomical variations	Free air, fat stranding, foreign bodies, +/− oral contrast	Use appropriate soft-tissue windows to enhance parenchymatous/intramural lesions. Change to lung for the detection of air	Examine during all contrast phases
Step 10: Vessels	Caliber, patency, stenosis, aneurysm, dissection, atherosclerosis, intramural hematoma, thrombus	Anatomical variations, collaterals, cavernous transformation, presence/absence of vessels	Intramural hematoma, contrast extravasation (active bleeding), perivascular fat stranding, intraluminal air	Use appropriate soft-tissue windows to enhance parenchymatous/intramural lesions. Change to lung for the detection of air	Examine during all contrast phases
Step 11: Nodes	Size (short axis), shape (lobular, irregular), presence of necrosis, homogeneity/heterogeneity, location	Anatomical variations, morphology (fatty hilum)	Assess for presence of necrosis	Use appropriate soft-tissue windows to enhance parenchymatous lesions	Examine during all contrast phases
Step 12: Peritoneum and retroperitoneum	Thickness of peritoneum, free air/fluid, mass lesions, hematoma, fat stranding, fibrosis, collections	Anatomical variations	Assess for presence of fluid/air, fat stranding	Use appropriate soft-tissue windows to enhance parenchymatous lesions. Change to lung for the detection of air	Examine during all contrast phases
Step 13: Bones	Mineralization, fractures, lytic/mass lesions, periosteal reaction, cortical destruction	Anatomical variations, bone cyst	Calcifications	Use bone window	Examine during all contrast phases
Step 14: Muscles	Atrophy/hypertrophy, asymmetry, mass lesions, invasion (tumor), hematoma, edema, fatty infiltration	Anatomical variations, morphology	Calcifications	Use appropriate soft-tissue windows	Examine during all contrast phases
Step 15: Air and fluid	Location (intraabdominal intraperitoneal, intraabdominal extraperitoneal, pseudoperitoneum), quantity, density, defects in the wall of a hollow viscus	Anatomical variations	Air, fluid	Use appropriate soft-tissue windows. Change to lung for the detection of air	Examine during all contrast phases
Step 16: Miscellaneous	Edema, abscesses, soft tissue defects/infiltration, tumors	Anatomical variations	Air, fluid, calcifications	Use appropriate soft-tissue windows. Change to lung for the detection of air	Examine during all contrast phases

## Data Availability

No new data were created.
